# Correction: Ryss, A.Y.; Subbotin, S.A. New Records of Wood- and Bark-Inhabiting Nematodes from Woody Plants with a Description of *Bursaphelenchus zvyagintsevi* sp. n. (Aphelenchoididae: Parasitaphelenchinae) from Russia. *Plants* 2023, *12*, 382

**DOI:** 10.3390/plants12162949

**Published:** 2023-08-15

**Authors:** Alexander Y. Ryss, Sergei A. Subbotin

**Affiliations:** 1 Zoological Institute, Russian Academy of Sciences, Universitetskaya Emb. 1, 199034 Saint Petersburg, Russia; alryss@gmail.com; 2 Plant Pest Diagnostic Centre, California Department of Food and Agriculture, 3294 Meadowview Road, Sacramento, CA 95832, USA; 3 Department of Entomology and Nematology, Hutchison Hall, University of California, Davis, CA 95616, USA; 4 Centre of Parasitology, A.N. Severtsov Institute of Ecology and Evolution, Russian Academy of Sciences, Leninskii Prospect 33, 117071 Moscow, Russia

In the original publication [1], there was missing information, namely a Zoobank registration link for a new species, *Bursaphelenchus zvyagintsevi* sp. n. The corrected main text appears below in Section 2.1:

*Bursaphelenchus zvyagintsevi* sp. n.

*Adults* (Figures 1 and 2, Table 2): http://zoobank.org/urn:lsid:zoobank.org:act:71BDE14D-B778-40FE-977D-D910F701DE6D (accessed on 13 August 2023). Body curved ventrally. Stylet base slightly expanded, but without distinct knobs. Cephalic annuli faintly distinct through light microscopy. Median bulb ellipsoid, large; valve median to sub-median of bulb. Excretory pore located at nerve ring or at posterior end of the median bulb. Lateral field with two incisures.

The authors state that the scientific conclusions are unaffected. This correction was approved by the Academic Editor. The original publication has also been updated.
