# Supplementary material for: New Records of Wood- and Bark-Inhabiting Nematodes from Woody Plants with a Description of Bursaphelenchus zvyagintsevi sp. n. (Aphelenchoididae: Parasitaphelenchinae) from Russia
Source: Plants (Basel). 2023 Jan 13;12(2):382. doi: 10.3390/plants12020382 (PMC9860568; doi:10.3390/plants12020382)
Supplement: Supplementary file 1 [file plants-12-00382-s001.zip › plants-2140119-supplementary.pdf]

Supplementary Materials

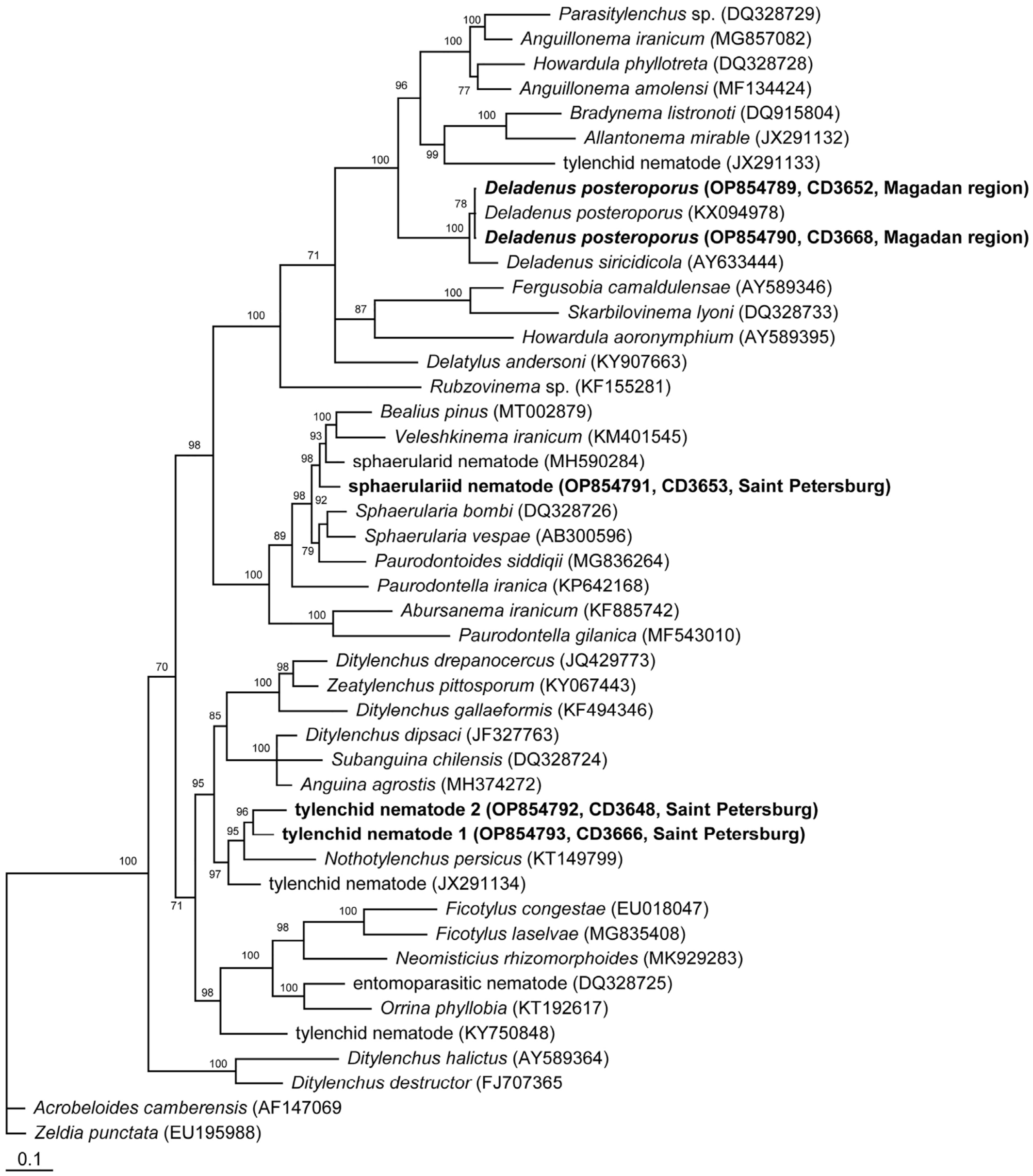

Figure S1: Phylogenetic positions of studied samples within some species from the order Tylenchida nematodes as inferred from Bayesian analysis of the D2-D3 of 28S rRNA (ntax=46 nchar=987) gene sequences. Posterior probability values more than 70% are given on appropriate clades. New sequences are indicated in bold.

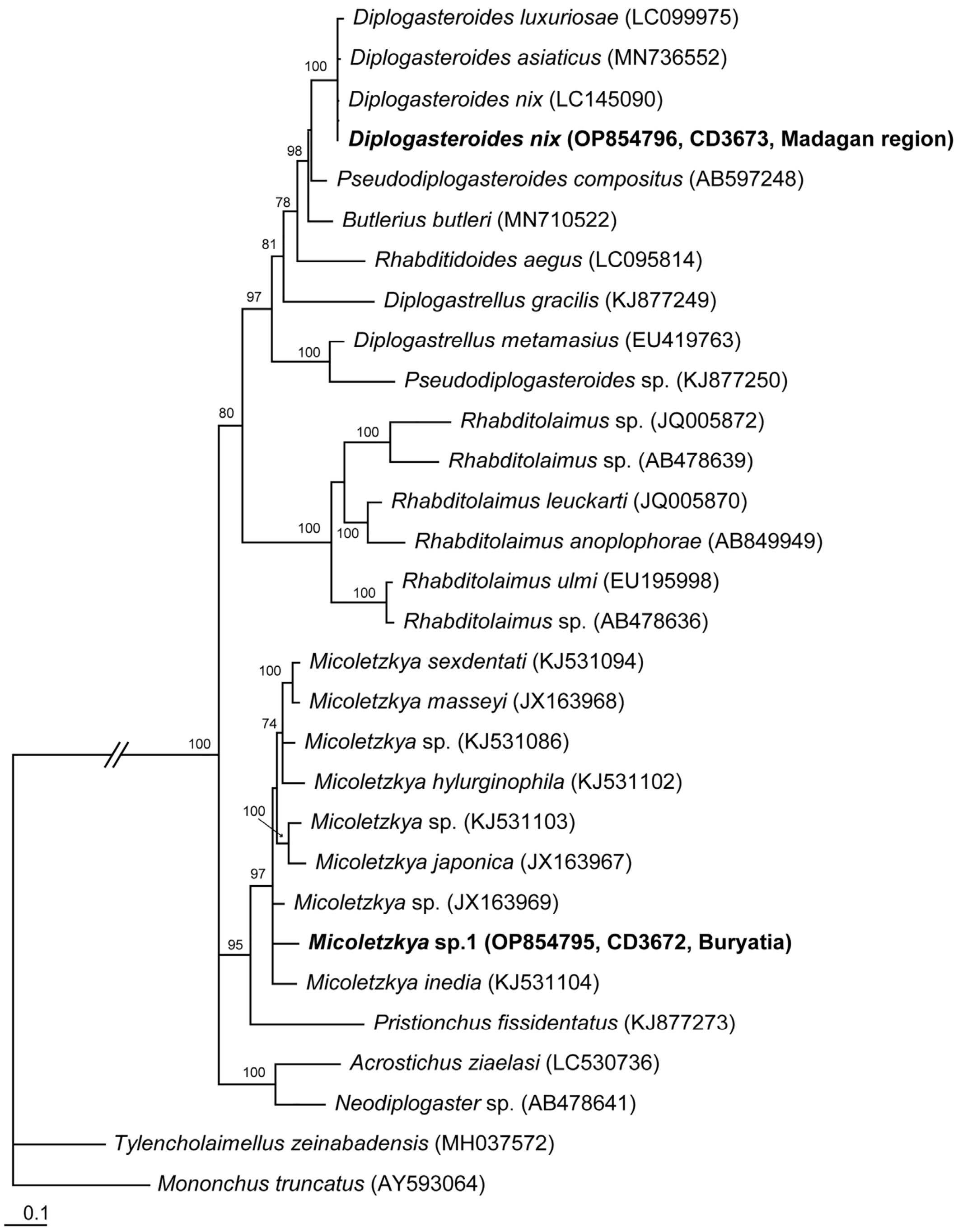

Figure S2: Phylogenetic positions of studied samples within some species from Diplogastridae as inferred from Bayesian analysis of the D2-D3 of 28S rRNA (ntax=31 nchar=794) gene sequences. Posterior probability values more than 70% are given on appropriate clades. New sequences are indicated in bold.

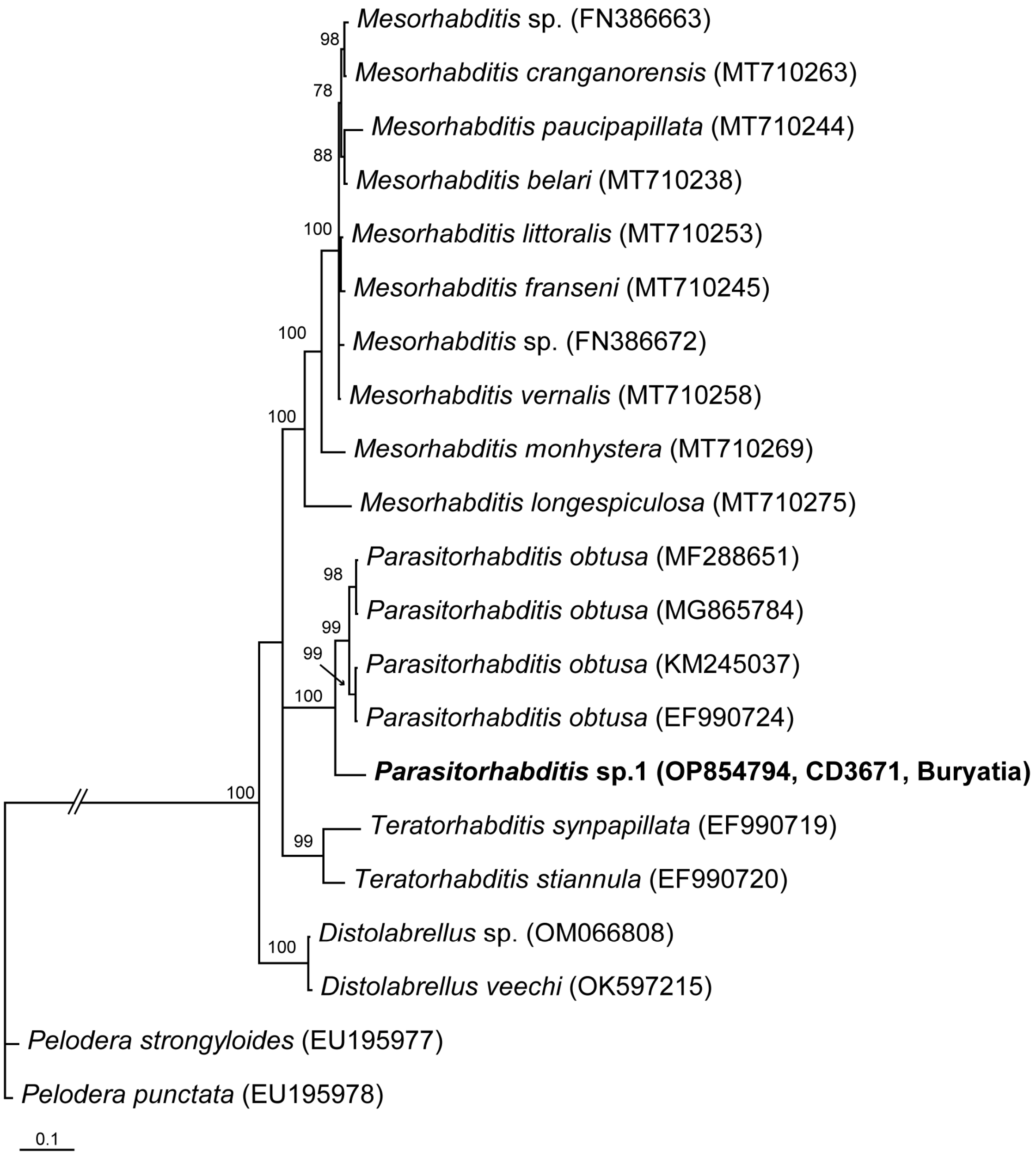

Figure S3: Phylogenetic relationships of *Parasitorhabditis* within some species from Rhabditidae as inferred from Bayesian analysis of the D2-D3 of 28S rRNA (ntax=21 nchar=576) gene sequences. Posterior probability values more than 70% are given on appropriate clades. New sequence is indicated in bold.

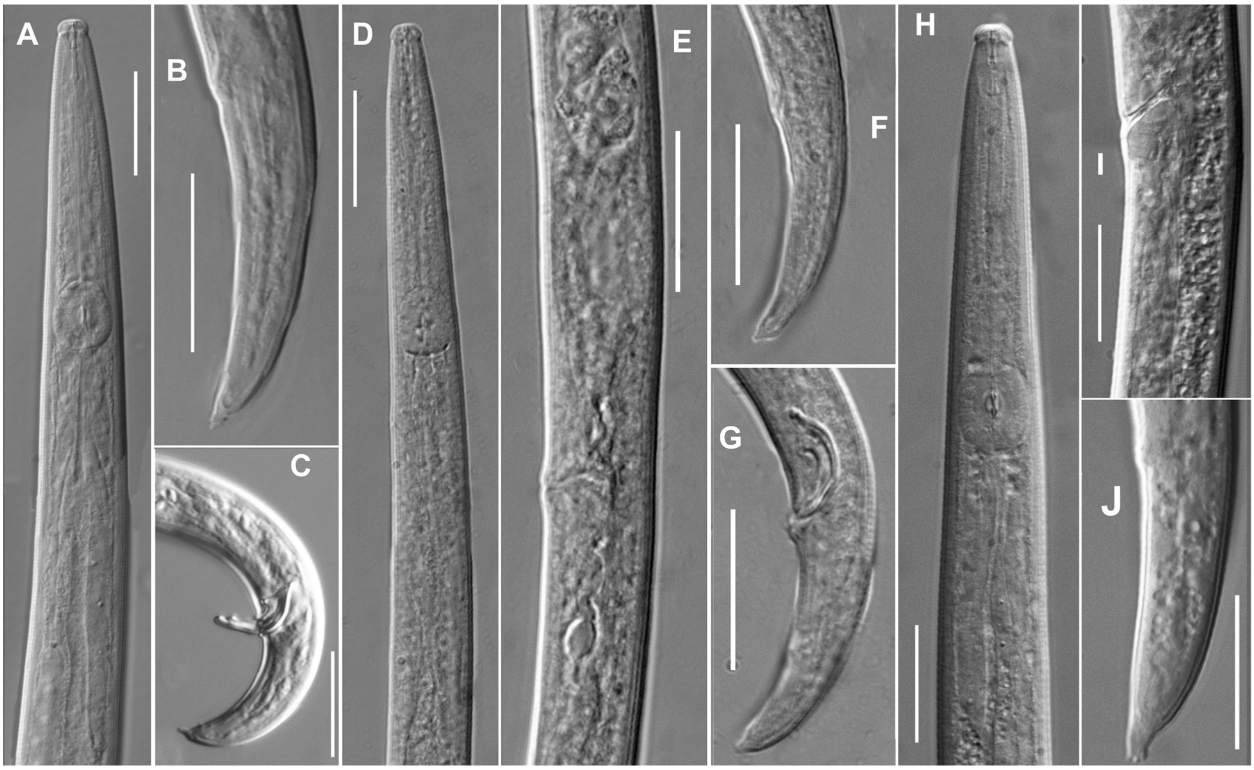

Figure S4: Anterior body end, male and female tails. A-C – *Aphelenchoides* sp.1 (CD3645); D-G – *Aphelenchoides heidelbergi* (CD3663); H-J – *Laimaphelenchus* sp1. (CD3674). Scale - 20 μm.

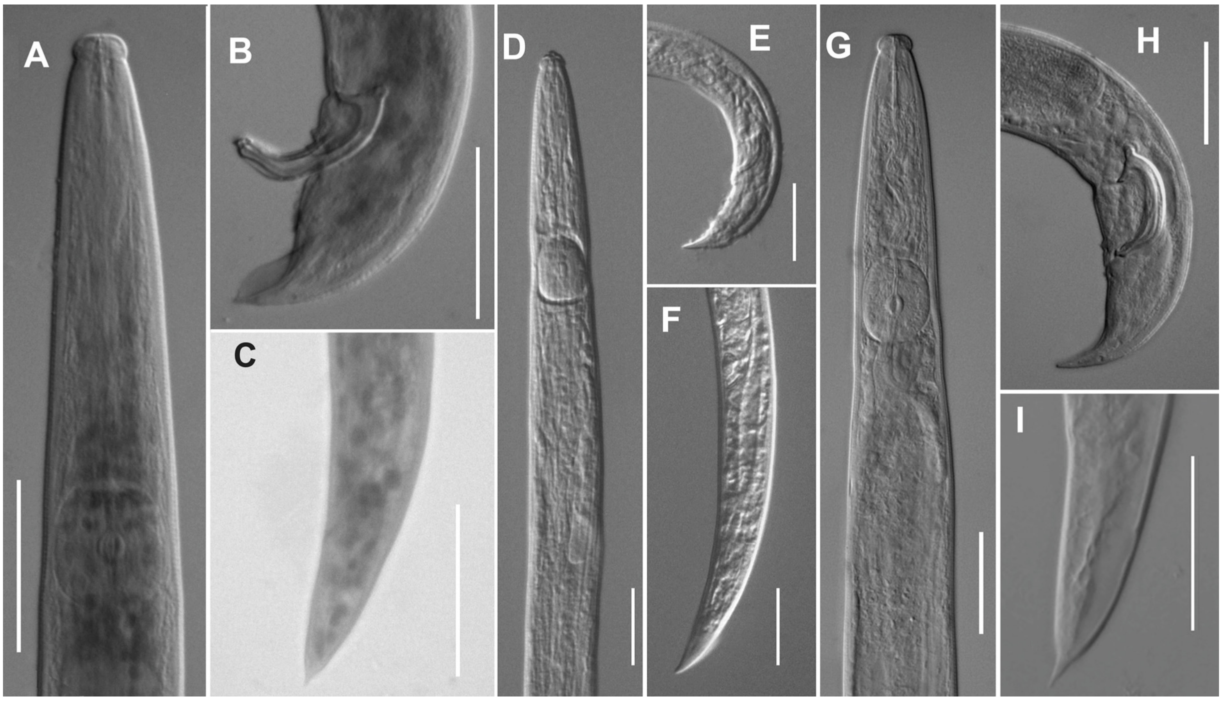

Figure S5: Anterior body end, male and female tails. A-C – *Bursaphelenchus fraudulentus* (CD3644); D-F – *B. eremus* (CD2936); G-I - *B. mucronatus* (CD3642). Scale - 20 μm.

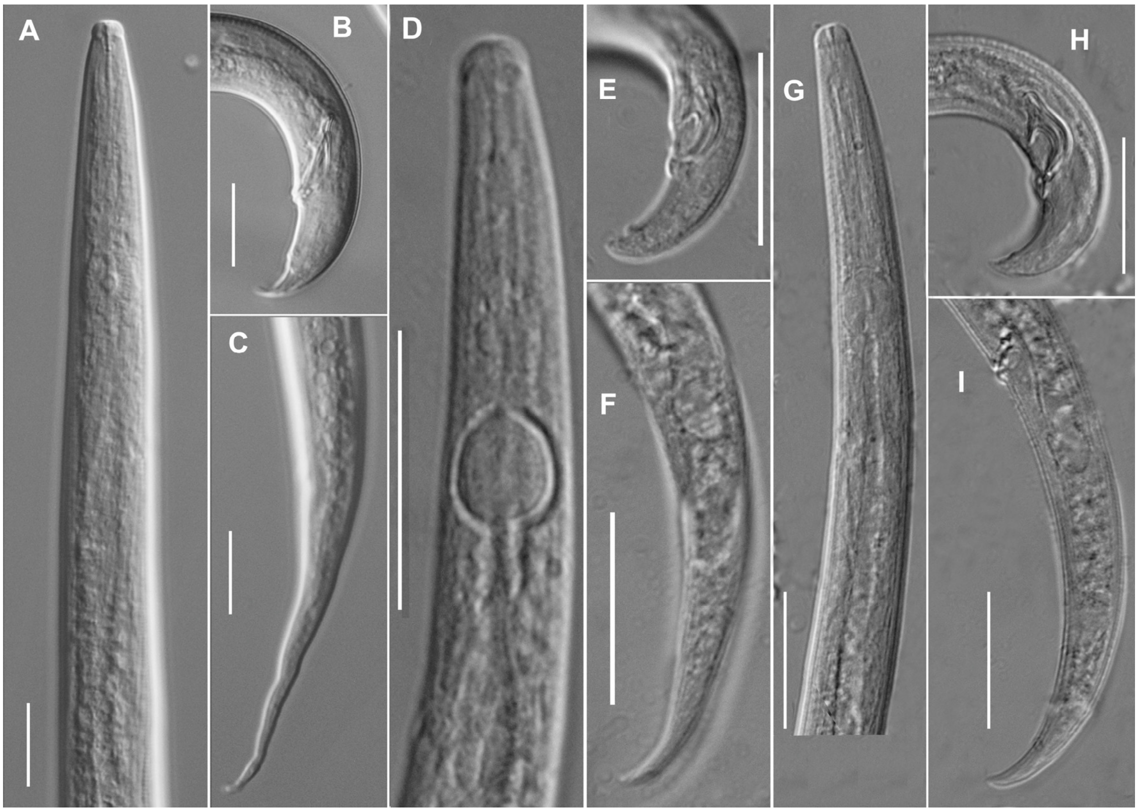

Figure S6: Anterior body end, male and female tails. A-C – *Bursaphelenchus willibaldi* (CD3664); D-F – *Cryptaphelenchus* sp.3 (CD3658); G-I – *Cryptaphelenchus* sp.1 (CD3655). Scale - 20 μm.
